# Ventriculosubgaleal shunt as a proposed technique for post-infectious hydrocephalus

**DOI:** 10.1007/s00381-022-05661-x

**Published:** 2022-10-10

**Authors:** Mohamed Mohsen Amen, Ahmed Zaher, Hatem Ibraheem Badr, Mohammad Fekry Elshirbiny, Ahmed Mahmoud Elnaggar, Amr Farid Khalil

**Affiliations:** 1 Neurosurgery Department, Mansoura University, Mansoura, Egypt; 2 Neurosurgery Department, Faculty of Medicine, Mansoura University, Mansoura, Egypt

**Keywords:** Post-infectious hydrocephalus, Ventriculosubgaleal shunt

## Abstract

**Background:**

The management of post-infectious hydrocephalus (PIH) remains challenging for neurosurgeons. It requires a temporary diversion procedure till the normalization of CSF parameters prior to the permanent one. Ventriculosubgaleal shunt (VSGS) was widely used in pediatric cases with post-hemorrhagic hydrocephalus (PHH). However, its role in PIH is still lacking. This study was done to elucidate the safety and efficacy of VSGS as a temporary CSF diversion procedure before the permanent one in patients with PIH.

**Patients and methods:**

This retrospective investigation analyzed the data of 50 consecutive cases who underwent VSGS for PIH.

**Results:**

The age of the included patients ranged between 1 and 10 months. Twenty-six cases had meningitis and or ventriculitis (52%), while the remaining had shunt infection. At follow-up, arresting of hydrocephalus was noted in ten patients (20%), while another 36 cases required the permanent diversion procedure within 35 days. Regarding the shunt complications, scalp infection, tissue breakdown, and shunt exposure were encountered in ten cases (20%), while CSF leakage was noted in 12 cases (24%). Shunt migration was noted in only two patients (4%). Shunt revision was needed in 16 cases (32%). Mortality was encountered in four cases (8%) because of sepsis. Risk factors for morbimortality included younger age, lower weight, male gender, and meningitis and or ventriculitis.

**Conclusion:**

VSGS is a safe and effective procedure in infants awaiting definitive VPS for postinfectious hydrocephalus. It was proven that VSGS has shortened the hospital stay and the economic burden on the country.

## Introduction

Hydrocephalus is a term describing an active enlargement of brain ventricles. This occurs secondary to impaired passage of cerebrospinal fluid (CSF) from its secretion site in the ventricles to its absorption site and then to the systemic circulation [1].

For about six decades, shunting CSF from the ventricles to another anatomical location, mostly the peritoneum, was the main management plan for such patients [2, 3]. However, some patients might need a temporary method of CSF diversion due to the presence of local or systemic infection, concomitant abdominal pathology hindering diversion to the peritoneum. These comorbidities must be corrected before insertion of the permanent shunt to decrease the rate of shunt infection [4–6].

These temporary methods include repeated transfontanellar tapping, external ventricular drainage (EVD), frequent lumbar drainage, Ommaya reservoir, and ventriculosubgaleal shunting (VSGS) [7, 8].

In the past, the application of VSGS was limited to neonates with intraventricular hemorrhage and consequent post-hemorrhagic hydrocephalus (PHH) [9, 10]. However, little is published regarding its application in pediatric patients with post-infectious hydrocephalus [11, 12].

The VSGS procedure entails creating an artificial shunt between the lateral ventricle and a pouch created in the subgaleal space, leading to the formation of a fluid collection of variable sizes. In contrast to other drainage methods, this approach offers a natural way for CSF absorption through the cervical lymphatics draining the subgaleal region [13].

We conducted the current study to elucidate the safety and efficacy of VSGS as a temporary CSF diversion procedure before VPS in patients with PIH, which is the most common cause of acquired hydrocephalus in our tertiary center.

## Patients and methods

This retrospective investigation was carried out at Mansoura University Neurosurgical Department following approval from the local scientific and ethical committee of our medical school. We retrospectively reviewed the data of 50 consecutive cases diagnosed with PIH as a consequence of meningitis, ventriculitis, or previous shunt infection that were managed by VSGS during the period between December 2019 and December 2021. We excluded patients who were managed by a temporary diversion procedure rather than the VSGS and patients with heavy CSF infection (purulent CSF), patients having brain abscesses and multiple interventricular septations.

All patients received standard preoperative care, including detailed history taking from the parents, neurological examination, and routine preoperative laboratory investigations, as well as radiological studies. All patients underwent MRI for diagnostic and follow-up purposes. Transcranial ultrasonography was done in some cases.

Anterior fontanelle tapping was done in all cases to obtain a CSF sample which was analyzed for the cellular count, type, protein and sugar content, gram stain, and culture. Prior to the surgical procedure, both the child’s parents signed a written consent after explaining the benefits and possible complications of the surgical procedure.

**Fig. 1:**
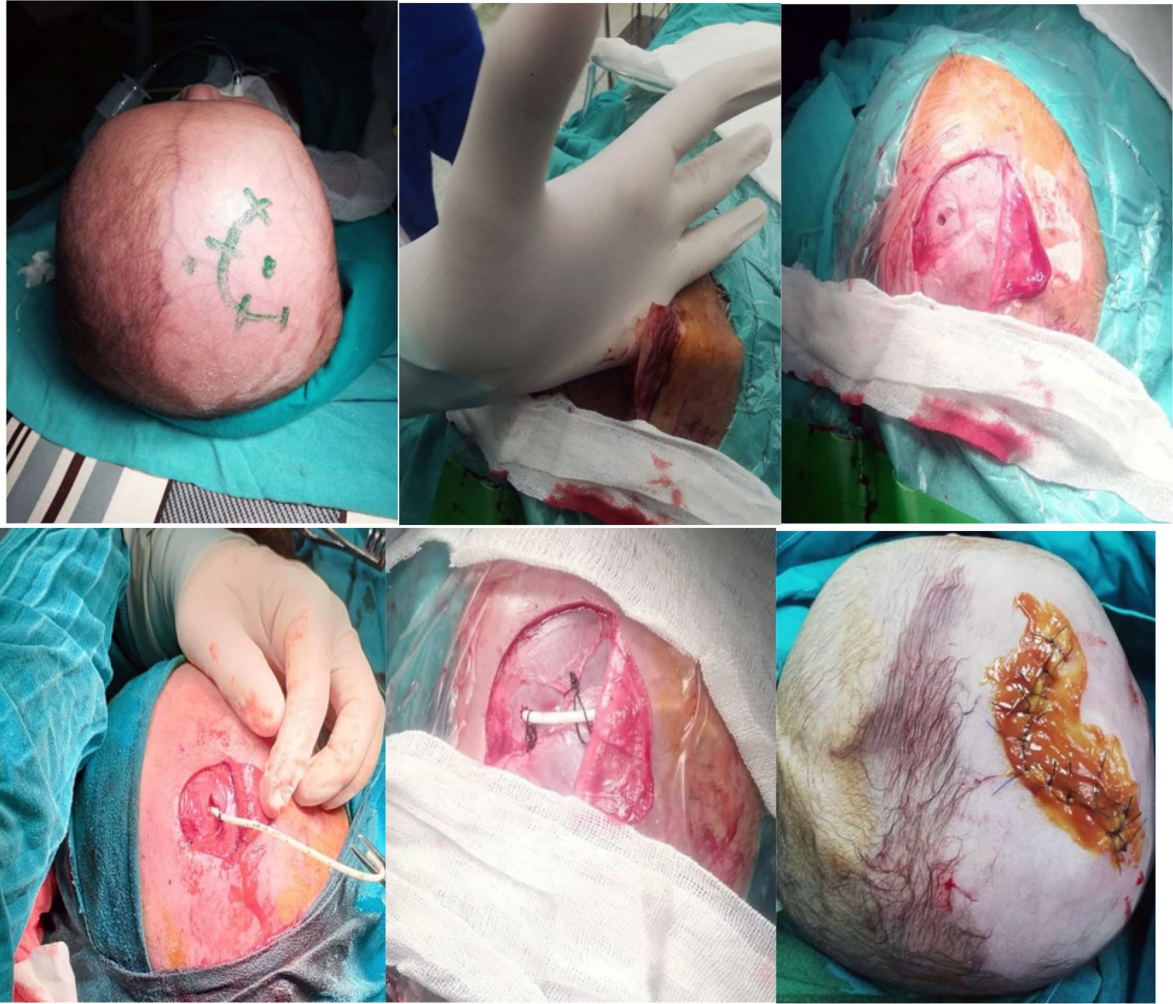
Operative steps

Regarding the surgical procedure (Fig. 1), we preferred to insert the VSGS through the lateral region of the anterior fontanelle. A curvilinear incision was performed over this region, and a skin flap was raised. Then, a large subgaleal pouch was created over the temporal and occipitoparietal regions, not involving the frontal region. A small bony opening was created anterior to the coronal suture about 3 cm lateral to the midline. After that, the dura was coagulated, followed by the insertion of the proximal part of the VSGS. After obtaining a CSF sample for analysis, the shunt was sutured to the periosteum by multiple sutures, followed by layered closure of the surgical wound.

**Fig. 2:**
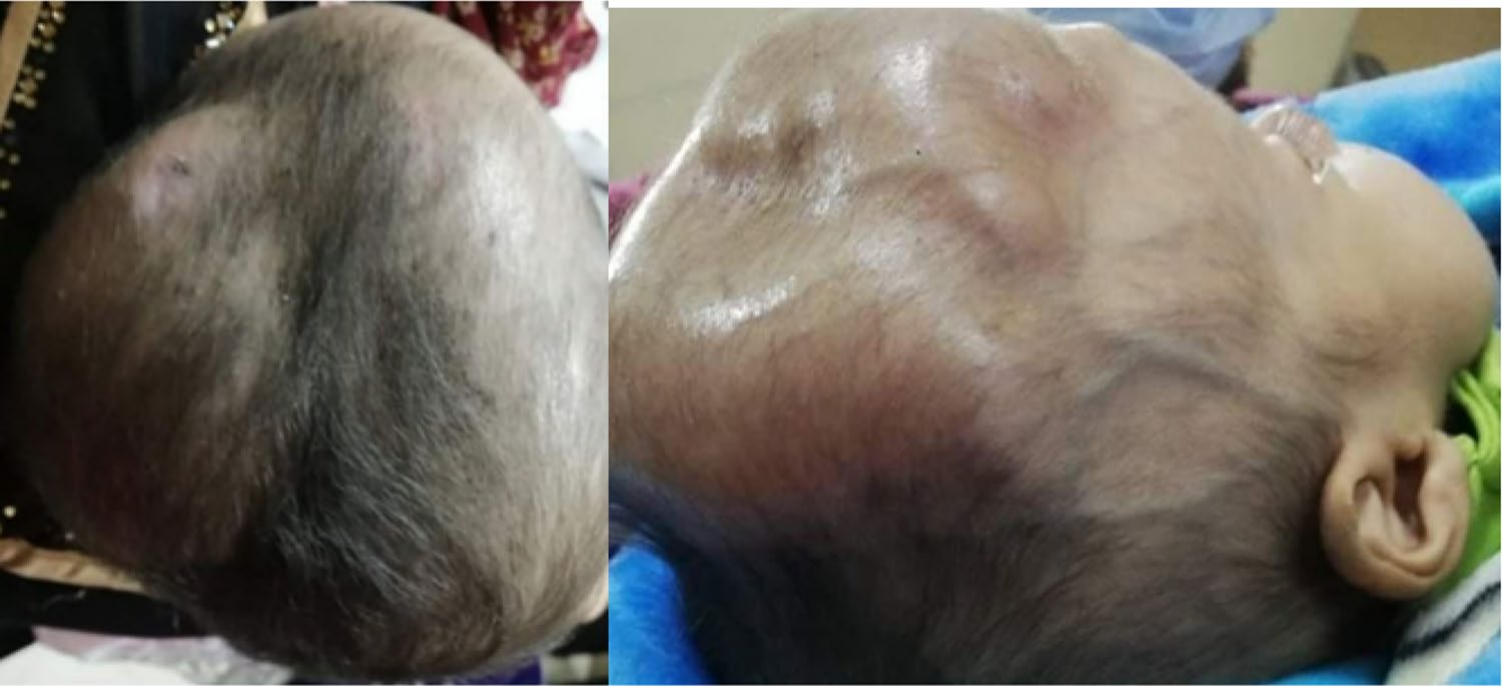
Post-operative view showing the distended subgaleal pouch

**Fig. 3:**
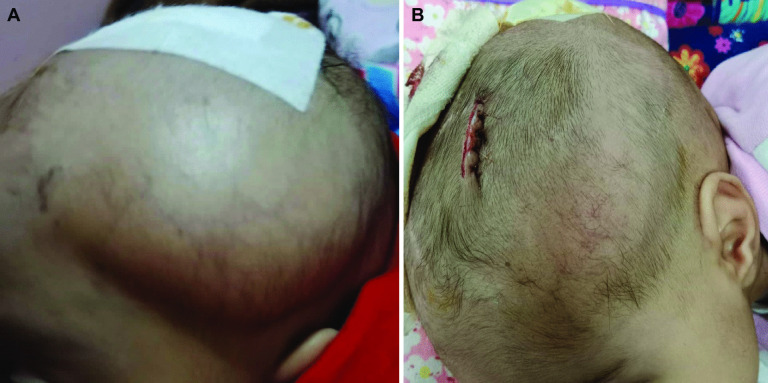
**A** After VSGS. **B** The same patient after shunt removal and inserting VPS

Post-operatively, patients were transferred to the recovery unit and then to the internal neurosurgical ward, where frequent monitoring was ensured. Patients were admitted 1 to 2 days to ensure presence of a pouch (Fig. 2); then, they were followed up weekly at OPC. Any complications, including infection, shunt exposure, CSF leakage, and shunt obstruction, were noticed, well managed, and recorded. When the subgaleal pocket becomes under-tension with no drainage of CSF, we began to perform repeated tapping from the pocket at regular intervals (4–7 days) under complete aseptic conditions. At the end of the tapping, we intended to leave some fluid inside the pouch to preserve its patency and prevent adhesion formation. Otherwise, when the VSGS was working well, CSF sample was taken for analysis and culture after 2 weeks from the procedure and the antibiotics were changed according to the updated culture results. Subsequently, regular CSF samples were taken weekly until CSF characteristics became optimal for VPS insertion (Fig. 3B). The time interval between the temporary and permanent diversion procedures was recorded.

The collected data were tabulated in Excel software for windows (Office 2019 version). Quantitative data were expressed as mean and range, while categorical data were expressed as frequency and percentage. The chi-square test or Fischer exact test was used to compare categorical data, while the Mann–Whitney test was used to compare numerical ones.

## Results

**Fig. 4:**
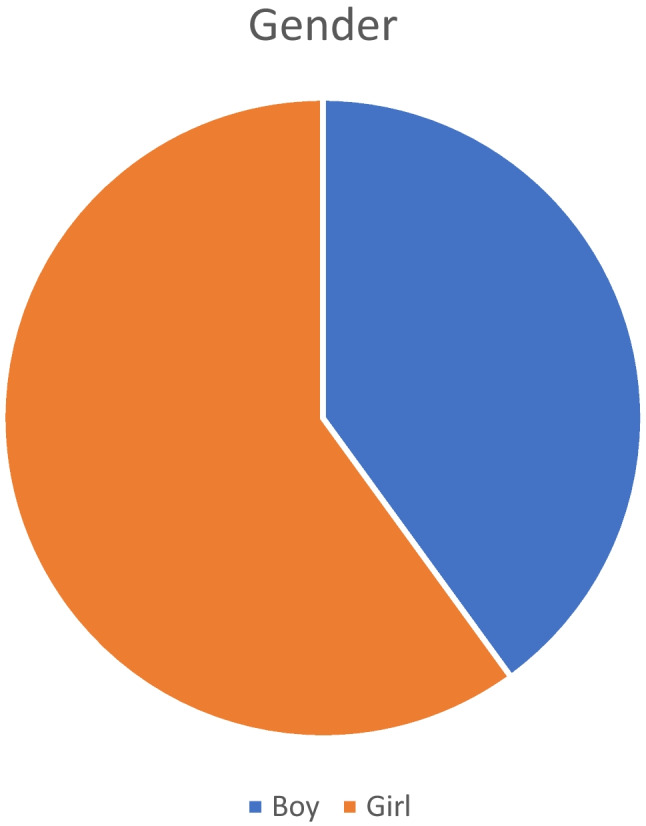
Gender distribution in the study population

**Fig. 5:**
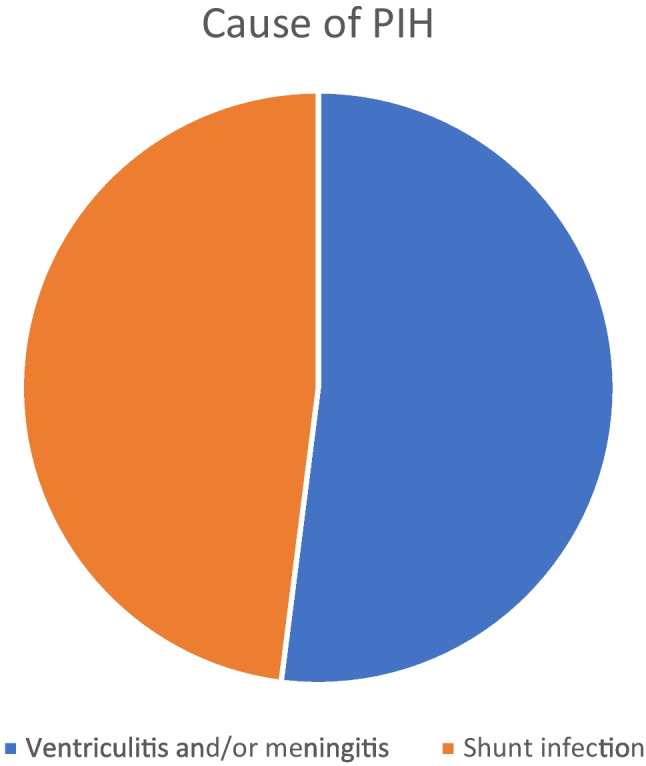
Etiology of PIH in the study population

The age of the included 50 patients ranged between 1 and 10 months (median = 3.5 months). Their weight had a median value of 6 kg. We included 20 boys (40%) in addition to 30 girls (60%). All of them had PIH either after meningitis and/or ventriculitis (26 cases—52%) or shunt infection (24 cases—48%) (Table 1, Figs. 4 and 5).

**Table 1:** Patient and disease criteria

**Item**	**Data**
**Age (months)**	3.5 (1–10)
**Weight (kg)**	6 (3–10)
**Gender**	
** -Boy**	20 (40%)
** -Girl**	30 (60%)
**Cause of PIH**	
** -Meningitis and/or ventriculitis**	26 (52%)
** -Shunt infection**	24 (48%)

Regarding preprocedural CSF characteristics, white blood cells ranged between 10 and 200, while glucose ranged between 20 and 40 mg/100 mL. In addition, protein level ranged between 60 and 450 mg/100 mL (Table 2).

**Table 2:** Preprocedural CSF characteristics of the study cases

**Item**	**Data**
**WBCs**	90 (10–200)
**Glucose (mg/100 mL)**	28 (20–40)
**Protein (mg/100 mL)**	160 (60–450)

**Fig. 6:**
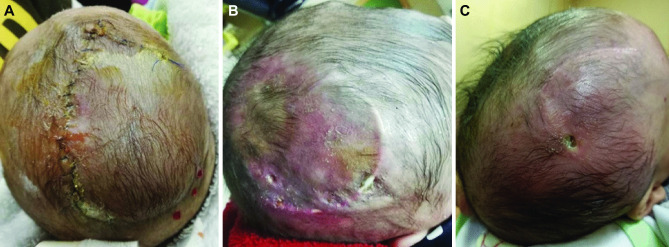
Complications following VSGS insertion. **A** CSF leak. **B** Shunt exposure. **C** Scalp infection, tissue breakdown and CSF leak

Regarding post-operative complications (Table 3 and Fig. 6), scalp infection, tissue breakdown, and shunt exposure were encountered in ten cases (20%), and they were managed by shunt removal and replacement in another site. Additionally, shunt obstruction was noted in four cases (8%) who also showed pouch collapse, and they were managed by shunt revision and repositioning on the contralateral side and replacement of the tube with a new one.

**Table 3:** Post-operative complications

**Item**	**Data**
**Infection, breakdown, and exposure**	10 (20%)
**CSF leakage**	12 (24%)
**Obstruction**	4 (8%)
**Migration**	2 (4%)
**Tapping for tense pocket**	15 (30%)

Limited CSF leakage from the wound incision without tissue breakdown or shunt exposure was noted in 12 cases (24%) that were managed by adding primary suturing to the leaking points which was enough to stop the leakage. Shunt migration was noted in only two patients (4%). Totally, shunt revision was needed in 16 cases (32%) due to infection, obstruction, or migration. Fifteen patients (30%) required tapping due to tense pocket.

Regarding the fate of VSGS, arresting of hydrocephalic manifestations clinically and radiologically was noted in ten patients (20%), while another 36 cases required the permanent VPS procedure within 35 days after the initial temporary one (range, 24–45 days). Mortality was encountered in four cases (8%) because of sepsis (Table 4).

**Table 4:** Fate of VSGS

**Item**	**Data**
**Conversion to VPS shunt**	36 (72%)
**Resolution (arrested hydrocephalus)**	10 (20%)
**Mortality**	4 (8%)

Morbimortality was encountered in 16 patients in the current study (32%). Risk factors for that outcome were younger age, lower weight, male gender, and previous meningitis or ventriculitis, as illustrated in Table 5.

**Table 5:** Risk factors for morbimortality in the study population

	**No Morbi/mortality** **(*n* = 34)**		**Morbi/mortality** **(*n* = 16)**		***P* value**
	***N***	**%**	***N***	**%**	
**Gender**					
**Boy**	**8**	**23.5**	**12**	**75**	0.001*
**Girl**	**26**	**76.5**	**4**	**25**	
**Meningitis/ventriculitis**	**10**	**29.4**	**16**	**100**	< 0.001*
**VPS infection**	**24**	**70.6**	**0**	**0**	
**Age**	**5 (1–10)**		**2 (1–7)**		0.001*
**Weight**	**6.5 (3–10)**		**4.5 (3–7)**		0.002*

*Statistically significant

## Discussion

PIH is still considered a major neurosurgical challenge in the pediatric population not only for its morbidity but also its management with shunting is more liable to infection and obstruction. Both of these consequences negatively impact the life of the child, and they are more encountered in premature babies [6, 14].

To avoid these dreadful consequences, the neurosurgeon should delay the permanent shunting procedure (like VPS) after the CSF become clear of infection, protein, and debris. During that period, a temporary method is needed to obtain CSF drainage. These methods are numerous, and the proper choice should depend on case severity and available resources [7, 15].

Theoretically, EVD should carry an increased risk of infection compared to the VSGS, as the former exposes the CSF to the external environment, and that was previously confirmed by multiple studies. This will lead to a substantial increase in the duration of hospitalization due to the higher need for drain repositioning along with the increased morbidity in an already ill neurosurgical patient [7, 16, 17]. Furthermore, repeated lumbar drainage is also associated with infectious complications and carries a risk of neural tissue injury [18].

In our institute, we used to manage pediatric cases with PIH via EVD or frequent transfontanellar tapping. Regarding transfontanellar tapping, it was associated with multiple visits, more brain atrophy, possibility of brain abscess formation and prolongation of the period of CSF infection. With regard to EVD, it was associated with longer hospitalization periods and high post-procedural newly acquired infections, as well as its high economic burden. To solve this dilemma, we intended to try another method of PIH management. In late 2019, we renewed our interest in VSGS and decided to apply it to patients with PIH.

This method was initially described by Mickulicz in 1896, and it was subsequently applied to 173 patients with hydrocephalus with different etiologies [19]. In addition, it was widely used in the temporary management of premature babies diagnosed with post-hemorrhagic hydrocephalus [20, 21]. Furthermore, the spectrum of VSGS has widened to manage tumor-associated hydrocephalus and PIH [6].

In our opinion, VSGS offers some advantages over other temporary methods when applied to PIH patients. It is not associated with fluid or electrolyte loss, and the closed drainage system does not expose the CSF to the external environment leading to a decreased infection risk. The time of the surgical procedure was also short (it can even be done at the bedside under completely sterile conditions), and the majority of cases were followed on an outpatient basis. This decreased the hospitalization period and associated healthcare costs.

For a long time, we believed that draining an infected CSF through a closed circuit, not externally, could prolong or worsen the preexisting infection. However, this misbelief was changed after reviewing the literature, which revealed good outcomes [6, 11, 30].

In the current study, the mean duration between the temporary and permanent shunt procedure was 35 days, after excluding cases that had arresting of their hydrocephalus. This duration is comparable with the one reported by Tubbs and his colleagues, which had a mean value of 37.4 days [6]. Other studies reported more prolonged durations like 53.9 and 56 days, as reported by Kariyattil et al. [12] who applied the same diversion in infective hydrocephalus patients, and Sil et al. [13] whom applied it in in variety of hydrocephalus in infants (including post-infectious and post-hemorrhagic types), respectively.

In the current study, we used a valveless tube for the shunting procedure, and we manually added some extra bores (by a syringe needle) to the shaft of the tube. We believe that these extra holes will guard against obstruction, especially in the presence of protein and cellular debris in the drained CSF. Although many studies have supported the use of valveless tubes, some neurosurgeons prefer to use tubes with valves and steady reservoirs for system fixation and repeated tapping in cases of pouch failure [13].

In our investigation, 16 cases (32%) required shunt revision to extend the life expectancy of the temporary shunt (due to infection, migration, or obstruction). In another study evaluating the role of VSGS in post-hemorrhagic hydrocephalus, at least one revision was needed in 35% of the included cases [22]. We followed the same surgical steps required for revision which were published by Tubbs et al. [23]. The wound incision over the old pocket was opened, and dissection was done within the subgaleal pocket about 270 degrees around the calvaria [23]. The dissection was continued laterally to at least a point connecting the superior aspect of the auricle after excluding the forehead. The drainage tube was usually changed, whereas the bony burrhole was changed if the overlying skin was severely infected or ulcerated.

Our findings showed the incidence of infection and exposure in 20% of our patients. Although some studies reported a low infection rates after VSGS (0–10%) [11, 24–27], other authors reported a high incidence rate (47.6%) [12]. One should keep in mind that the majority of these studies were conducted in the PHH population rather than the PIH ones, apart from the one conducted by Kariyattil et al. [12], which included infective cases and reported a higher rate compared to ours. We think that a 20% incidence rate is an acceptable rate especially after we reviewed the archives of our patients who underwent an EVD insertion and found a higher rate of infection which reached to 24%. Moreover, one should consider that the operation is performed in an already contaminated field.

In the current study, the date of the permanent VPS was scheduled when the protein content of the CSF decreased to the normal range, and the bacterial culture was negative. Of note, we inserted the VPS through the same burrhole of the previous VSGS. If the skin overlying that point was ulcerated or heavily infected, a new burrhole was created in the occipitoparietal region either on the ipsilateral or contralateral sides.

In the current study, CSF leakage was encountered in 12 cases (24%), and that lies within the reported rate of CSF leakage from the surgical site after VSGS, which lies between 4.7 and 32% [23, 24, 26, 28, 29].

Our findings showed that shunt obstruction and migration were detected in 8% and 4% of our cases, respectively. Sil et al. reported that the same complications were noticed in 15% and 2% of their 215 participants who had hydrocephalus of different etiologies, including the PIH [13].

In our study, mortality was encountered in only four patients (8%) because of sepsis. Mortality rates following VSGS range between 9 and 20% [12, 30], and most of these cases died because of other complications rather than shunt-related ones [23, 26].

Our findings showed arresting of the hydrocephalus in ten patients (20%). The cases were clinically and radiologically free from any manifestations of increased ICP. Reports of hydrocephalus resolution after VSGS are rare. Alan and his associates reported a 9.1% resolution rate of post-hemorrhagic hydrocephalus, as only two out of the included 22 cases did not require the permanent VPS [22]. Although the incidence of arrested hydrocephalus is still low after such a procedure, its predictors should be studied in larger trials to specify this treatment modality for patients who are expected to show resolution.

Our findings showed that younger age and lower weight were significant risk factors for morbimortality after VSGS, and we think that might be attributed to the increased skin thickness and maturity of lymphatic channels with advancing age.

Our study has some limitations in the form of the relatively small sample size collected from a single neurosurgical institution. Also, long-term follow-up data are lacking. These drawbacks should be well handled in the upcoming studies.

## Conclusion

Based on the previous findings, VSGS could be a safe and efficacious option for temporary CSF diversion in patients with PIH before the permanent VPS, especially in resource-limited settings. It is associated with an acceptable complication rate.

## Data Availability

All data related for this study are available for sharing upon request.
